# HIV, Stigma, and Rates of Infection: A Human Rights and Public Health Imperative

**DOI:** 10.1371/journal.pmed.0040052

**Published:** 2007-01-30

**Authors:** Susan Timberlake, Jason Sigurdson

In their essay “HIV, Stigma, and Rates of Infection: A Rumour without Evidence”, Daniel Reidpath and Kit Yee Chan rightly underscore the insufficient body of research on the relationship between stigma and discrimination and HIV transmission [1]. Increased scientific attention and effective programming against stigma and discrimination are both sorely needed. But the Joint United Nations Programme on HIV/AIDS (UNAIDS) does not accept a number of points made in the essay.

Discrimination based on health status, including HIV, is a human rights violation, and stigma is the social form of this violation. HIV stigma and discrimination are wrong in and of themselves, and should be stopped for that reason alone. Reidpath and Chan suggest, as “an alternative hypothesis to the UNAIDS position”, that stigma against certain groups, including people living with HIV, may have a public health value because it “could reduce opportunities for contact between high- and low-risk groups”. UNAIDS cannot endorse a hypothesis that bases a public health goal on a human rights violation; nor do we believe it is either right, or necessary, to pit the public health against human rights.

The right to health—and human value, dignity, and autonomy, the bases of human rights—requires that people have the information, services, and support they need to protect their health and avoid causing harm to others. So does the public health. In this context, people living with HIV should not and need not be social pariahs to achieve public health goals; neither should HIV-negative people be put in a position of “protecting themselves” through stigma against others.

UNAIDS stands by the view that stigma and discrimination increase vulnerability to both infection and the impact of HIV. People, including those living with HIV, repeatedly make clear that their fear, or real experience, of stigma and discrimination affects whether, when, and how they take up HIV prevention and treatment goods and services. Thus, efforts to get people to protect themselves from getting infected, or if infected, to find out about their status, prevent the onward transmission of HIV, and access treatment, are hindered by stigma and discrimination.

UNAIDS encourages both governments and researchers to focus on what Reidpath and Chan consider “the more-difficult issues relating to the manner in which HIV spreads in populations, the social vulnerabilities it exploits, and the ways in which individuals…interact with each other”. But the complexities the authors cite undermine their argument that stigma between groups may result in a reduction of HIV transmission. In dealing with HIV, it has become very clear that people's behaviour transcends population groups over time and space. The belief that risk rests in “the other” creates a false perception of safety and may in fact increase vulnerability.

Stigma and discrimination by governments against particular groups, such as women, the poor, sex workers, ethnic minorities, people who use drugs, migrants, men who have sex with men, and prisoners, often means that those most affected by HIV receive the least attention in the response. This makes the national HIV response less effective and compounds human rights violations against these people.

Contrary to the authors' assertion that the link between stigma and the epidemic has “become the basis for considerable policy and program development”, very little in fact is being done about stigma and discrimination in national programmatic responses. This is cause for great concern, given that stigma and discrimination have been identified as major barriers to achieving universal access to prevention, treatment, care, and support in all the consultations on the subject. Will governments finally implement programmatic responses to overcome stigma and discrimination? We believe it is essential that they do so.

As the number of infections increase and millions still need treatment, UNAIDS welcomes the growing body of research on HIV stigma and discrimination. Among other things, we are supporting people living with HIV to develop an index on stigma and discrimination. We hope that data from this tool will both provoke and assist governments to overcome stigma and discrimination in their national responses to HIV. People and governments confronting HIV should do so not out of fear or stigma, but out of the knowledge that we all share the same rights and protecting these rights protects us all from HIV and AIDS.

## References

[pmed-0040052-b001] Reidpath DD, Chan KY (2006). HIV, stigma, and rates of infection: A rumour without evidence. PLoS Med.

